# Highly Efficient Adsorption of Heavy Metals and Cationic Dyes by Smart Functionalized Sodium Alginate Hydrogels

**DOI:** 10.3390/gels8060343

**Published:** 2022-05-31

**Authors:** Tianzhu Shi, Zhengfeng Xie, Xinliang Mo, Yulong Feng, Tao Peng, Dandan Song

**Affiliations:** 1 Department of Brewing Engineering, Moutai Institute, Renhuai 564500, China; xinliangmo@163.com (X.M.); fengyulong520110@163.com (Y.F.); edifcztony@126.com (T.P.); vi_veneto@163.com (D.S.); 2 Oil & Gas Field Applied Chemistry Key Laboratory of Sichuan Province, College of Chemistry and Chemical Engineering, Southwest Petroleum University, Chengdu 610500, China; xiezhf@swpu.edu.cn

**Keywords:** sodium alginate, modification, heavy metals, dye, adsorption

## Abstract

In this paper, functionalized sodium alginate hydrogel (FSAH) was prepared to efficiently adsorb heavy metals and dyes. Hydrazide-functionalized sodium alginate (SA) prepared hydrazone groups to selectively capture heavy metals (Pb^2+^, Cd^2+^, and Cu^2+^), and another functional group (dopamine grafting), serves as sites for adsorption methylene blue (MB), malachite green (MG), crystal violet (CV). Thermodynamic parameters of adsorption indicated that the adsorption process is endothermic and spontaneous. The heavy metals adsorption by FSAH was physical adsorption mainly due to *ΔH*^θ^ < 40 kJ/mol, and the adsorption of cationic dyes fitted with the Langmuir models, which indicated that the monolayer adsorption is dominated by hydrogen bonds, electrostatic interactions, and π-π interactions. Moreover, the adsorption efficiency maintained above 70% after five adsorption-desorption cycles. To sum up, FSAH has great application prospect.

## 1. Introduction

In the process of rapid industrial development, it is easy to cause environmental pollution problems. The improper treatment of industrial waste water leads to the destruction of the water ecological environment [1]. In particular, heavy metals and dyes in sewage are very resistant to biodegradation, posing a serious threat to human health [2], for example, lead poisoning can cause brain damage and kidney and liver dysfunction; excessive malachite green can also cause nausea, abdominal pain, etc. [3,4,5,6,7]. The adsorption method is widely used due to its advantages of low-cost, simple-operation, and good selectivity. Heavy metals and dyes coexist in wastewater, and the current adsorption material treatment capacity limits its large-scale application [8,9,10,11]. Researchers are committed to developing adsorbents with large adsorption capacity, no secondary pollution, and wide application.

SA, a natural polysaccharide, is widely used in the food industry, pharmaceutical industry, rubber industry, and other industries. At the same time, SA as adsorption material to adsorb heavy metals and organic pollutions through physical and chemical modification, has attracted wide attention [12,13]. The chemical modification of SA can be divided into: the C–C bond of *o*-diol that was oxidized to dialdehyde or dicarboxylic groups; and COO^–^ on SA was grafted to new groups, such as amides or esters [14]. 

Hydrazone is very sensitive to heavy metals and can easily coordinate to form coordination structures, [15,16,17,18]. DA (dopamine) has strong functional modification ability, and catechol and amine groups could form various hydrogen bonds to adsorb target pollutants [19,20,21]. DA-modification is a highly functional surface modification strategy. Combining the advantages of the hydrazone group and DA-modification, an SA-based adsorption material FSAH was prepared. 

In this paper, the functional modification of sodium alginate was divided into two steps: the hydrazone group was prepared by using sodium alginate and biphthalate dihydrazide (BDD) based on the Schiff base reaction, and DA was grafted to form an amide carbonyl group to prepare the adsorbent FSAH. The adsorption capacity of heavy metals and dyes was evaluated, and pH, initial concentration, time, and reaction temperature were investigated. The adsorption isotherm equations, adsorption thermodynamics, and adsorption kinetics were analyzed in the adsorption process. According to FT-IR, SEM, and XPS analysis, the mechanism of FSAH adsorption of heavy metals and dyes was proposed. Adsorption–desorption experiments showed FSAH had broad application prospects in the removal of heavy metals and dyes.

## 2. Results and Discussion

### 2.1. Characterizations

#### 2.1.1. SEM and EDS–Mapping Analysis

**Figure 1:**
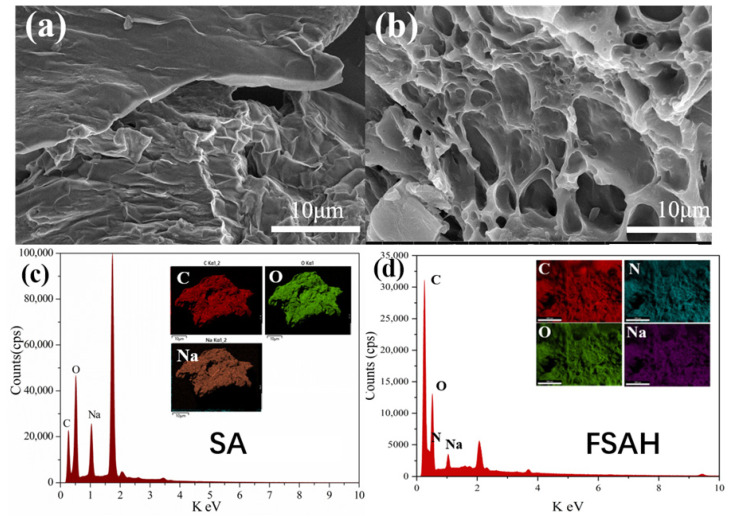
SEM of SA (**a**) and FSAH (**b**); EDS–mapping of SA (**c**), FSAH (**d**).

The microstructure and morphology of material before and after modification were recorded by SEM, respectively. The SA surface was smooth with dense small particles (Figure 1a), but the surface of FSAH (Figure 1b) showed rough, multi-fractured, and porous structure resulting from SA that was grafted with BDD and DA, respectively, and the specific surface area (BET) was increased. Figure 1c,d shows the C, O, and Na elements in SA by EDS–mapping as shown in Table S1, but the N element is added to FSAH after modification. The element content of C increased from 32.49 to 48.35%, however, O and Na contents decreased, respectively. The new element N increased to 25.88%, as shown in Table S5.

#### 2.1.2. FT-IR and XPS Analysis

**Figure 2:**
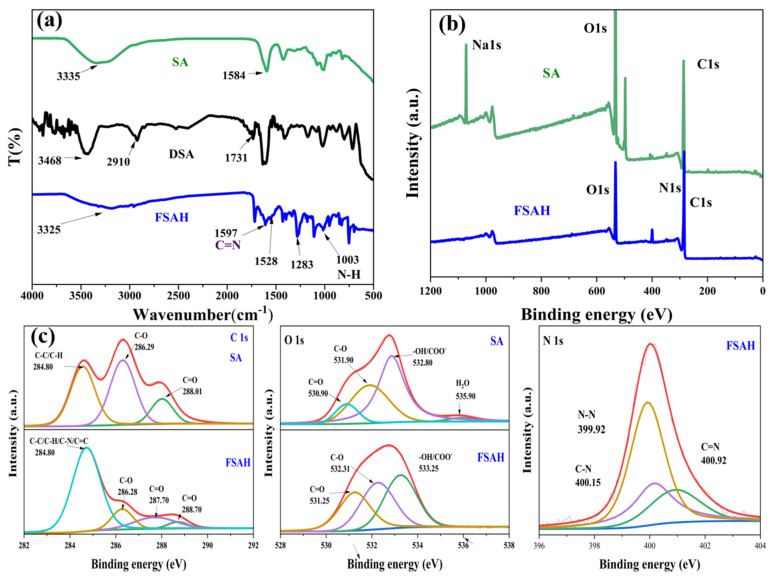
(**a**) FT–IR spectra of SA, DSA, and FSAH. (**b**) The XPS spectra of SA and FSAH. (**c**) C 1s, O 1s, and N 1s spectra of SA and FSAH.

The infrared absorption spectra of FSAH, DSA (dialdehyde sodium alginate), and SA were determined using FT-IR spectroscopy (Figure 2a). The dialdehyde group (1731 cm^−1^) was generated by oxidation of the C–C bond in *o*-diol groups by NaIO_4_, and the stretching vibration of the aldehyde group proton was observed at 2910 and 2842 cm^−1^ of DSA [22]. In FSAH, the C=N imine and the N-H peak occurred at 1597 and 1003 cm^−1^, respectively, and the 1731 cm^−1^ peak vanished, indicating that the hydrazone group was successfully prepared [23]. The connection between *δ* _N-H_ and *ν* _C-N_, as well as the C=N peak positions, created the amide II (1528 cm^−1^) and amide III (1283 cm^−1^) belts peaks, respectively.

As shown in Figure 2b,c, C 1s, O 1s, and N 1s core levels were investigated using deconvoluted fitting of complex spectra, and the N 1s, which was new peak, occurred in FSAH. The C 1s spectrum for SA displayed the bonds as being C–C, C–H bond (284.80 eV), O–C–O bond (286.29 eV), C–OH bond (286.88 eV), and C=O bond (288.01 eV), respectively [24,25]. However, a new peak of 287.70 eV appeared in C 1s of FSAH, belonging to the C=N bond in the hydrazone group, and the other three peaks BE shifted to 284.80, 286.28, and 288.70 eV, successively. The atom fraction at 284.80 eV increased to 70.47%, indicating that the dopamine grafting was successful. The peak of 535.90 eV in FSAH disappeared, the electron binding energy of –OH bond increased from 532.80 to 533.25 eV in O 1s, and the atomic fraction of the –OH decreased by 19.32% (from 55.96% to 36.64%). The N 1s curve formed by the Schiff base and aldimine condensation reactions, with BEs of 399.92, 400.15, and 400.92 eV for the C=N, N–N, and C–N bonds, respectively. The structure of the planned target adsorbent FSAH is compatible with these results.

#### 2.1.3. TG and BET Analysis

**Figure 3:**
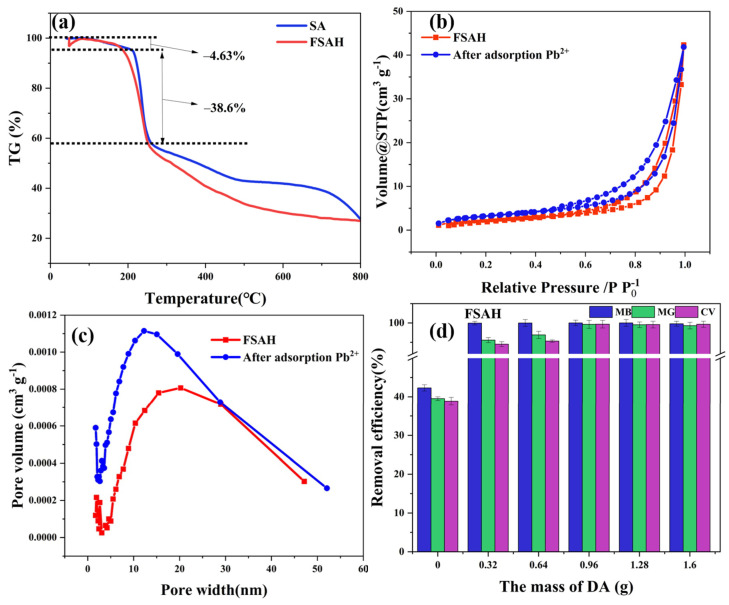
The TG curves of FSAH (**a**), SA BET N_2_ adsorption and desorption isothermals curve for FSAH and FSAH after adsorption Pb^2+^ (**b**,**c**), the relationship between different DA mass ratios and dye removal rates (**d**).

As Figure 3a shows, the decomposition of SA and FSAH involved three processes: mass loss of 9% was attributed to the loss of water at around 210 °C in the first step; the thermal decomposition weight loss rate of carboxyl, hydrazone, amide, and hydroxyl groups increased clearly under 260 °C; and the breakdown and decomposition of the molecular carbon chain and basic skeleton (T > 260 °C). 

Figure 3b,c showed that N_2_ adsorption increases slowly under relatively low pressure, the adsorption capacity of N_2_ increased obviously with the increase of pressure which can be classified as *VI* type adsorption isotherm according to IUPAC classification criteria, which showed a lag loop of H3 associated with slit pores when the relative pressure (P/P_0_) is close to 0.6. After the adsorption of Pb^2+^ by FSAH, the average pore size of BJH decreased, and the concentrated distribution of pore size changed from 20.34 to 12.29 nm. The pore uniformity was good, but the volume of adsorption pores of BET and BJH increased greatly.

### 2.2. Adsorption Performance Study

#### 2.2.1. DA Mass Ratio

The DA was applied to prepare FSAH to adsorb cationic dyes (MB, MG, and CV) with variable proportions (1.60, 1.28, 0.96, 0.64, and 0.32 g). When the amount of DA increased from 0 to 0.96 g, the removal rates of MB, MG, and CV increased from 42.31, 39.52, and 38.85 to 99.99, 99.61, and 99.66% (Figure 3d). The removal efficiency of cationic dyes did not change significantly when the amount of DA was increased. This indicated the carboxyl group on the SA chain was conjugated to a certain amount of DA by N-(3-dimethylaminopropyl)-N′-ethylcarbodiimide hydrochloride (EDC), and N-hydroxysuccinimide (NHS), and excessive DA remained in the PBS buffer. Considering cost and benefit, the input of DA grafting was determined to be 0.96 g.

#### 2.2.2. pH

**Figure 4:**
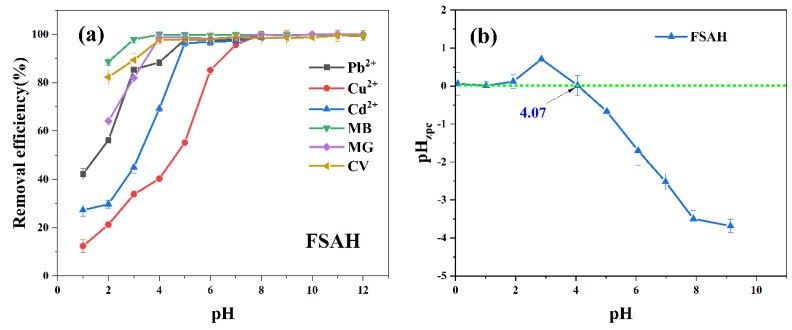
The relationship between pH and heavy metals and dyes removal rate (**a**); the pH_ZPC_ of FSAH (**b**).

The relationship between pH and heavy metals (pH: 1–7) and dyes (pH: 1–12) removal rate is shown in Figure 4. The point of zero charge (pH_ZPC_) value of FSAH at zero potential was 4.07. The amount of H^+^ was increased, causing protonation of the adsorbent surface at pH < pH_ZPC_, resulting in a positively charged surface. At pH > pH_ZPC_, the level of OH^–^ was increased, and functional groups of FSAH surfaces formed negative charge, thus promoting the removal rate of heavy metals and cationic dyes [26]. The pH of 5 was the best for FSAH to adsorb heavy metals and cationic dyes, because of the hydroxide formation at pH > 5; as Medusa software showed formation of copper hydroxide at pH = 6; and then the adsorption removal rate had no significant changes to dyes at pH > 5. When pH < pH_PZC_, the adsorption ability was restricted because a mass of H^+^ on FSAH surface caused group protonation, which prevented the adsorption removal rate [27,28].

#### 2.2.3. Adsorption Isotherm 

To evaluate the equilibrium adsorption mechanism, the Langmuir [29], Freundlich, and Redlich–Peterson isotherm models [30] were used (Equations (S1)–(S4), respectively).

**Figure 5:**
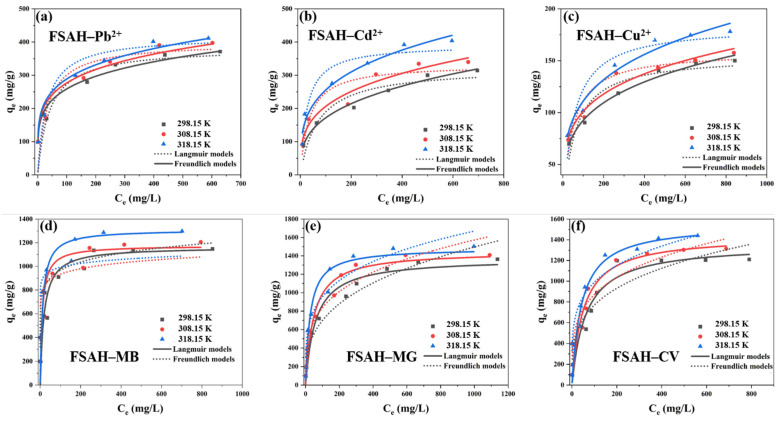
FSAH adsorption isotherm curves fitted by the Langmuir and Freundlich models to adsorb Pb^2+^ (**a**), Cd^2+^ (**b**), Cu^2+^ (**c**), MB (**d**), MG (**e**), and CV (**f**).

Compared with the fitting correlation coefficient (*R*^2^) of the Freundlich isotherm model and Langmuir isotherm model, the Freundlich isotherm model was more suitable to describe the adsorption process of Pb^2+^, Cd^2+^, and Cu^2+^ by FSAH at three temperature conditions in Figure 5 (*R*^2^ > 0.95). The adsorption intensity decreased with the increase in temperature (1/*n* < 0.5) and *K_F_* increased with the increase of temperature, which indicated that the adsorption process was easier to proceed with accompanied by an increase in temperature. In the Langmuir isotherm model, Figure 5 shows the change curve of adsorption separation factor (*R_L_*) and adsorbent initial concentration, 0 < *R_L_* < 1, indicating that the adsorption behavior is favorable and has a strong affinity (Figure S1). It can be inferred that the adsorption process of heavy metal ions mainly tended to be multilayer adsorption. In the adsorption process of MB, MG, and CV, the fitting correlation coefficient *R*^2^ of the Langmuir isotherm model was >0.9, which was larger than the Freundlich isotherm model’s *R*^2^, and the adsorption data were more consistent with the Langmuir isotherm model. These results indicated that the adsorption of MB, MG, and CV by FSAH mainly involved monomolecular adsorption induced by the point-facing adsorption mechanism. *K_F_* and *n* represented the adsorption strength and strength, respectively. The *K_F_* and *n* values of MB were the largest when fitting parameters with the three cationic dyes, which indicated that the affinity of FSAH with cationic dyes was MB > CV > MG [31]. Rendering a high *R*^2^ value and a low χ^2^ value, fitting with the Redlich–Peterson model was also fairly good (*R*^2^ > 0.9, Table S2). The saturated adsorption capacity of Pb^2+^, Cd^2+^, Cu^2+^, MB, MG, and CV by FSAH were 371.4, 304.3, 157.1, 1147.71, 1332.75, and 1210.01 mg/g in Table S1, respectively. Comparison with other adsorption materials are reported in Table S6. The adsorption capacity of FSAH on heavy metals and dyes has a relatively large advantage. As a consequence, FSAH was a valuable adsorbent material for wastewater treatment.

#### 2.2.4. Adsorption Kinetic

In order to fit the experimental data, the non-linear pseudo-first-order (PFO) [32], pseudo-second-order (PSO) [33] rate laws kinetic models, and internal diffusion models are represented by Equations (S5)–(S7) in this paper, respectively.

**Figure 6:**
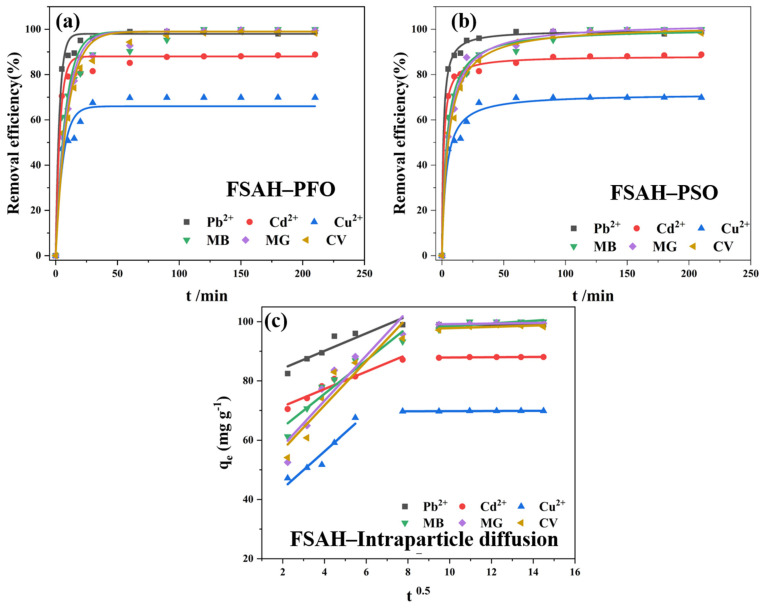
The PFO (**a**) and PSO kinetic plots (**b**), the intraparticle diffusion plots (**c**) for heavy metals and cationic dyes onto FSAH.

Figure 6 and Tables S2 and S3 show that the adsorption of heavy metal ions and cationic dyes had a higher degree of fit with PSO, *R*^2^(PSO) > *R*^2^(PFO) and χ^2^(PSO) < χ^2^(PFO) based on high values of *R*^2^ and low values of the non-linear chi-square statistics (χ^2^), and the *q_e_* calculated by PSO was more consistent with the actual value of the experiment. The relevant kinetic fitting parameters *K*_1_, *K*_2_ > 0, which showed that the adsorption behavior of heavy metals was spontaneous. In the case of the coexistence of chemical adsorption and physical adsorption, the rate-determining step was chemical adsorption. *C*_1_, *C*_2_ ≠ 0 in the two stages of intraparticle diffusion adsorption, indicating that boundary layer diffusion has a great influence on adsorption. The *k_id_*_1_ > *k_id_*_2_ which indicated that the surface of the adsorbent was occupied by the adsorbate with the increase of adsorption time, and the adsorbate molecules diffused from the surface of the adsorbent to the internal pores of the adsorbent, resulting in a very slow process and a decrease in the adsorption efficiency [34].

#### 2.2.5. Thermodynamic Adsorption

**Figure 7:**
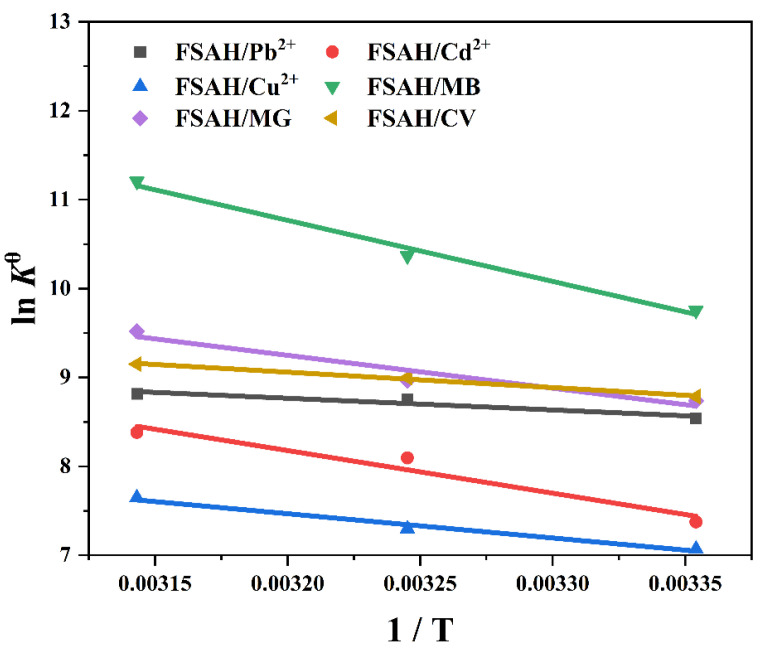
The adsorption capacity and the adsorption thermodynamics curve of heavy metal ions and dyes adsorption of FSAH at 298.15, 308.15, and 318.15 K, respectively.

The Gibbs free energy (*ΔG^θ^*), enthalpy (*ΔH^θ^*), and entropy (*ΔS^θ^*) were calculated using Equations (S8)–(S11) at 298.15, 308.15, and 318.15 K, respectively [35,36]. Figure 7 and Table S4 show the related thermodynamic parameters. The *ΔH* > 0 indicated that increasing the temperature increased the resultful collision efficiency between heavy metals, dyes, and FSAH, which promoted diffusion in the microchannels inside, improved adsorption ability to adsorb heavy metals which were very advantageous to adsorption. The *ΔS* > 0 showed that solute adsorption was accompanied by solvent desorption in the adsorption process; the former process was accompanied by a loss in entropy, whereas the latter process increased entropy [37]. At various temperatures, the *ΔG^θ^* < 0, adsorption processes were viable and spontaneous. In conclusion, adsorption reactions were spontaneous endothermic reactions.

#### 2.2.6. Reuse Adsorption 

**Figure 8:**
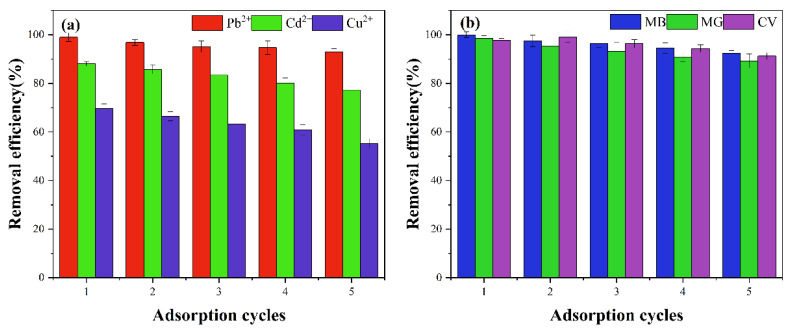
Removal efficiency of FSAH (**a**,**b**) adsorption of heavy metals and dyes after 5 recycles.

To assess recycle adsorption ability of FSAH was an important index [38]. After five recycles, the adsorption removal rate of FSAH decreased to 93.15%, 77.28%, 55.18%, 92.45%, 89.23%, and 91.23% for Pb^2+^, Cd^2+^, Cu^2+^, MB, MG, and CV (Figure 8), respectively. The main reasons were speculated on the partial functional groups that were occupied with heavy metals and dyes incomplete desorption, and the structure of FSAH could be damaged with HCl [39]. In general, the FSAH as heavy metals and dyes adsorbent materials possessed excellent regeneration and reuse potential.

### 2.3. Adsorption Mechanism

**Figure 9:**
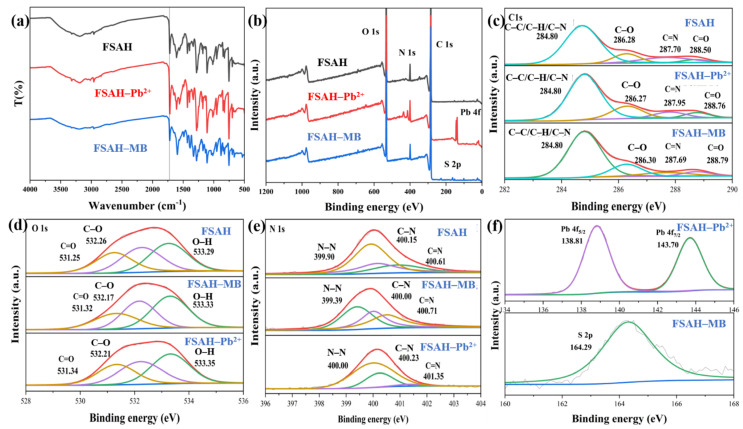
FT–IR (**a**) and XPS (**b**) analysis of the FSAH, and XPS analysis (**c**,**d**) of C 1s, O 1s, and N 1s (**e**) of the FSAH before and after adsorption of Pb^2+^ and MB, XPS analysis of Pb 4f and S 2p (**f**), respectively.

Figure 9a shows the FT-IR analysis of FSAH before and after adsorption of Pb^2+^ and MB. The peak of C=N came from Schiff base condensation shifted from 1597 to 1586 cm^−1^, The peak of COO^–^ at 1392 cm^−1^ changed initial position, which showed that ion-exchange and coordination would have occurred after adsorption Pb^2+^. The peak of carboxyl groups shifted significantly after adsorption MB, and new peaks appeared.

The new peaks of Pb 4f and S 2p clearly emerged in Figure 9b revealed that the FSAH had adsorbed Pb^2+^ and MB from XPS analysis, respectively. The O 1s peak for C–O of –COO^–^ shifted from 532.60 to 532.83 eV which possibly revealed the covalent interaction between Pb^2+^ and C–O of –COO^–^ was ionic interactions after adsorption Pb^2+^ [40,41,42]; however, the BE of C=N shifted from 401.28 to 402.05 eV (ΔBE > 0.5 eV), the atomic fractions of C=N were significantly reduced which indicated the preferential covalent interaction between Pb^2+^ and N-donor ligands over O-donors [10,43,44,45]. After adsorption of MB on FSAH, the S 2p_3/2_ (163.90 eV) and S 2p_1/2_ (168.60 eV) appeared in Figure 9c, and the O 1s spectra noticeably shifted, possibly from the influence of –OH and COO^–^ in FSAH. Because the dyes of MB, MG, and CV have positive-charge, FSAH has negative-charge when the system pH is greater than pH_ZPC_, and the adsorption data of MB, MG, and CV fitted with the Langmuir model which showed that monolayer adsorption was dominant. 

In general, the most possible explanation for adsorption is the ion exchange interaction from COO^–^ groups and chelation coordination from hydrazone groups played important roles in adsorption of heavy metals in FSAH; due to graft DA introducing functional groups that have more adsorption effect on dyes, especially catechol groups, which utilized electrostatic attraction, hydrogen bonding, π-π interaction, and Van der Waals force [46].

## 3. Conclusions

The secondary grafting of hydrazide and DA to prepare adsorption material FSAH, which has a high removal rate for Pb^2+^, Cd^2+^, Cu^2+^, MB, MG, and CV. The saturated adsorption capacity of FSAH for Pb^2+^, Cd^2+^, Cu^2+^, MB, MG, and CV were 371.4, 304.3, 157.1, 1147.71, 1332.75, and 1210.01 mg/g, respectively. SEM, BET, FT-IR, and XPS analysis show that heavy metals are easier to enter the FSAH in the spatial network structure; the structure of BDD boosted adsorption effectiveness of dyes through the π–π-interaction. At ideal pH = 5, the thermodynamic study revealed that the adsorption process is endothermic and spontaneous. The hydrazone groups coordination and ion exchange, which was primarily chemical adsorption, fitted the Freundlich model for heavy metal ion adsorption. However, cationic dye adsorption was linked to a variety of interactions, including H–bond, electrostatic, and π-π interaction, which fitted the Langmuir model and revealed that monolayer adsorption was the most common. Furthermore, after fivefold adsorption–desorption, adsorption efficiency can still be over 80%. To sum up, we concluded that FSAH was a valuable adsorbent material for wastewater treatment.

## 4. Materials and Methods

### 4.1. Materials

SA (500–1000 mPa·s) was purchased from Adamas Reagent (Shanghai, China). Acetic acid, BDD, dopamine (DA), ethylene glycol, Pb (NO_3_)_2_, Cd (NO_3_)_2_·4H_2_O, Cu (NO_3_)_2_·4H_2_O, MB, MG, CV, EDC, NHS, lead, cadmium, and copper standard solution (1000 mg/L) were obtained from Aladdin Biochemical Technology (Shanghai, China). Anhydrous ethanol, and sodium periodate (NaIO_4_) were obtained from the Kelong Chemical Reagent Factory (Chengdu, China). Unless otherwise noted, all reagents were used without further purification.

### 4.2. Preparation of DSA

SA (5 g) was dispersed in anhydrous ethanol (50 mL) for 2 h, and 40 mL aqueous solution (2.5 g, NaIO_4_) was dropped into SA-ethanol dispersion in the dark for 12 h (318.15 K, pH = 4.0). Furthermore, ethylene glycol (5 mL) was used to terminate reaction, the product was washed 3 times with a mixture of ethanol and water (v/v = 5:4), and finally, DSA was vacuum-dried [47]. Using an automatic potentiometric titrator and the hydroxylamine hydrochloride method, the oxidation degree (OD) of DSA was determined by potentiometric titration for the detection of aldehyde groups [22,37], and the OD of DSA is 64.28%.

### 4.3. Preparation of FSAH

**Figure 10:**
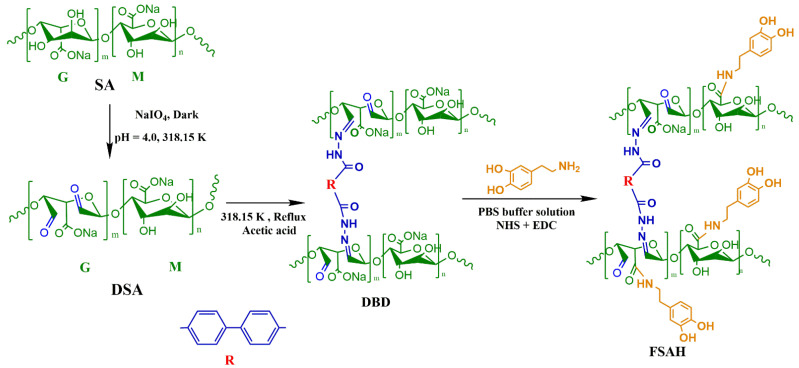
The preparation route of FSAH.

The mixture (DSA, 5.00 g and BDD, 2.70 g) was stirred to prepare DBD at 318 K for 12 h in ethanol (20 mL), which was collected by filtration; DBD (500 mg), EDC (440 mg), and NHS (295 mg) were stirred in PBS buffer solution (50 mL) for 30 min, and then added to 900 mg DA through nitrogen protection for 24 h at 15 °C. The hydrogel particle of FSAH was washed 3 times, and freeze-dried [18,21]. The preparation route of FSAH is presented in Figure 10.

## Data Availability

Not applicable.

## Supplementary Materials

The following supporting information can be downloaded at: https://www.mdpi.com/article/10.3390/gels8060343/s1, Figure S1: the change curve of adsorption separation factor *R_L_* [29,30,48]. Table S1: Parameters calculated by Langmuir and Freundlich models for heavy metals and dyes adsorption onto FSAH (298.15 K, 308.1 5K, 318.15 K) [29,30]. Table S2. Kinetic parameters for heavy metals and cationic dyes adsorption onto FSAH [32,33,49]. Table S3. Intraparticle diffusion parameters for heavy metals and cationic dyes adsorption onto FSAH. Table S4. Thermodynamic parameters for the adsorption of heavy metal ions and dyes onto FSAH [35,36]. Figure S2. Effect of different salt ions on the adsorption capacity of FSAH for to adsorb Pb^2+^ (a), Cd^2+^ (b), Cu^2+^ (c), MB (d), MG (e), and CV (f) [50,51,52,53,54]. Table S5. EDS-mapping parameters of SA and FSAH, respectively. Table S6. Comparison with other adsorption materials [10,31,55,56,57,58,59,60,61,62,63,64,65,66].
