# Supplementary material for: Highly Efficient Adsorption of Heavy Metals and Cationic Dyes by Smart Functionalized Sodium Alginate Hydrogels
Source: Gels. 2022 May 31;8(6):343. doi: 10.3390/gels8060343 (PMC9222840; doi:10.3390/gels8060343)
Supplement: Supplementary file 1 [file gels-08-00343-s001.zip › gels-1743557-supplementary.pdf]

gels

Supplementary Materials

Highly Efficient Adsorption of Heavy Metals and Cationic Dyes by Smart Functionalized Sodium Alginate Hydrogels

Tianzhu Shi [1,2,*], Zhengfeng Xie [2], Xinliang Mo [1], Yulong Feng [1], Tao Peng [1] and Dandan Song [1]

[1] Department of Brewing Engineering, Moutai Institute, Renhuai 564500, China; xinliangmo@163.com (X.M.); fengyulong520110@163.com (Y.F.); edifcztony@126.com (T.P.); vi_veneto@163.com (D.S.)
[2] Oil & Gas Field Applied Chemistry Key Laboratory of Sichuan Province, College of Chemistry and Chemical Engineering, Southwest Petroleum University, Chengdu 610500, China; xiezhf@swpu.edu.cn
* Correspondence: shitianzhu1018@163.com; Tel.: +86-18586420308

Adsorption isotherm

At equilibrium, the adsorption isotherm represents the effect between the amount of adsorbent per unit mass and solute adsorbed and left in the liquid phase [1]. To evaluate the equilibrium adsorption mechanism, the Langmuir [2] and Freundlich isotherm models [3] were used. Equations (S1) and (S2) typically presents the Langmuir models.

$$q_e = \frac{q_m K_L C_e}{1 + K_L C_e} \qquad \text{(S1)}$$

$$R_L = \frac{1}{1 + K_L C_0} \qquad \text{(S2)}$$

where q_e(mg/g) represents the amounts of adsorbent removal at equilibrium state, q_m(mg/g) is the maximum monolayer adsorption capacity, K_L (L/mg) represents the constant of affinity between adsorbent and adsorbate, R_L represents the Langmuir equilibrium dimensionless parameter, and C_e (mg/L) represents the adsorbate concentration after adsorption. The Freundlich models is presented by Equation (S3):

$$q_e = K_F C_e^{1/n} \qquad \text{(S3)}$$

where n and K_F (mg/g) represents the adsorption intensity and Freundlich constant of the experimental adsorption process, respectively.

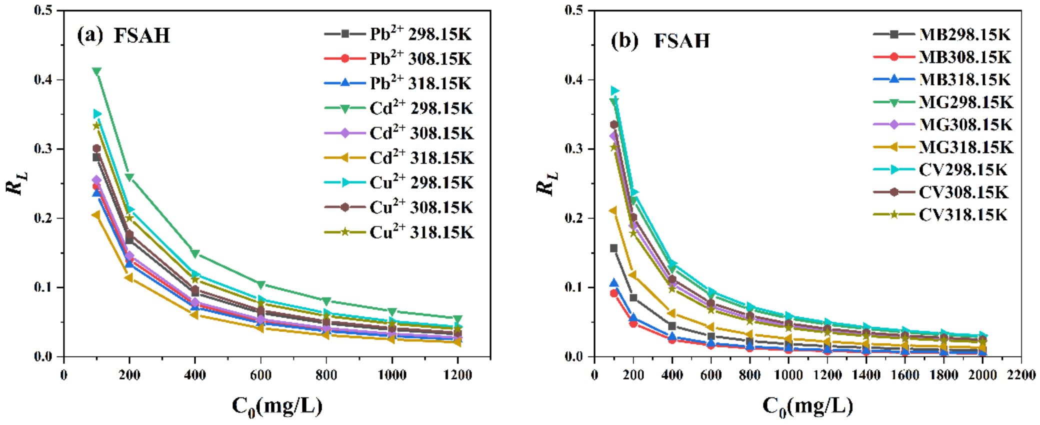

Figure S1. the change curve of adsorption separation factor R_L of adsorption heavy metals (**a**) and cationic dyes (**b**).

Table S1. Parameters calculated by Langmuir and Freundlich models for heavy metals and dyes adsorption onto FSAH (298.15K, 308.15K, 318.15K).

FSAH		Langmuir Models				Freundlich Models		
	T(K)	q_m(exp, mg g^{-1})	q_m(cal, mg g^{-1})	K_L	R^2	K_F	$1/n$	R^2
Pb^{2+}	298.15	371.4	383.1	0.02470	0.893	104.008	0.1981	0.969
	308.15	397.6	398.7	0.03063	0.902	111.723	0.1972	0.967
	318.15	411.7	417.7	0.03254	0.896	115.446	0.1992	0.976
Cd^{2+}	298.15	304.3	323.1	0.01420	0.891	39.667	0.3184	0.987
	308.15	340.1	332.6	0.02916	0.906	55.905	0.2835	0.953
	318.15	404.2	391.8	0.03886	0.935	71.012	0.2781	0.969
Cu^{2+}	298.15	157.1	154.3	0.01851	0.967	30.237	0.2429	0.985
	308.15	161.6	158.9	0.02324	0.909	36.701	0.2203	0.958
	318.15	180.2	183.6	0.01999	0.898	36.003	0.2448	0.971
MB	298.15	1147.71	1163.88	0.05380	0.957	611.121	0.0998	0.901
	308.15	1184.07	1176.39	0.09938	0.937	676.771	0.0699	0.883
	318.15	1298.51	1310.47	0.08448	0.934	802.255	0.0463	0.899
MG	298.15	1332.75	1372.10	0.01709	0.966	152.159	0.3311	0.912
	308.15	1407.75	1449.39	0.02136	0.971	236.277	0.2744	0.927
	318.15	1481.01	1483.69	0.03741	0.967	314.237	0.2418	0.910
CV	298.15	1210.01	1258.14	0.01602	0.989	219.625	0.2725	0.888
	308.15	1312.38	1338.24	0.01983	0.957	275.898	0.2497	0.898
	318.15	1439.37	1453.56	0.02306	0.925	362.205	0.2182	0.896

Adsorption kinetic

Application of adsorption kinetics to study adsorption rate and adsorption mechanism [4]. In order to fit the experimental data, the linear pseudo-first-order (PFO) [5], pseudo-second-order (PSO) [6] rate laws kinetic models and internal diffusion models are represented by Equations (S4)–(S6) in this paper, respectively:

$$\ln(q_e - q_t) = \ln q_e - k_1 t \quad \text{(S4)}$$

$$\frac{t}{q_t} = \frac{1}{k_2 q_e^2} + \frac{t}{q_e} \quad \text{(S5)}$$

$$q_t = k_{id}\, t^{0.5} + C \quad \text{(S6)}$$

where q_e and q_t (mg/g) are denoted adsorption uptake for heavy metal ions at equilibrium and at any time t (min), whereas k_1 (min^{-1}), k_2 (g mg^{-1} min^{-1}) are denoted the rate constants of PFO and PSO, respectively. k_{id} (mg g^{-1} $min^{-1/2}$) is denoted the intraparticle diffusion rate constant, C is denoted as the constant of boundary layer thickness.

Table S2. Kinetic parameters for heavy metals and cationic dyes adsorption onto FSAH.

Adsorbent			PFO			PSO		
		q_e(exp)	q_e(cal)	k_1	R^2	q_e(cal)	k_2	R^2
FSAH	Pb^{2+}	98.98	91.16	4.02×10^{-2}	0.9278	99.21	1.008×10^{-2}	0.999
	Cd^{2+}	88.05	82.61	4.24×10^{-2}	0.9045	89.28	1.12×10^{-2}	0.999
	Cu^{2+}	69.71	49.89	9.34×10^{-2}	0.8328	71.53	1.39×10^{-2}	0.999
	MB	99.99	91.28	1.63×10^{-2}	0.912	102.04	0.98×10^{-2}	0.999
	MG	99.25	87.21	2.14×10^{-2}	0.809	100.60	9.94×10^{-2}	0.999
	CV	98.37	88.76	2.93×10^{-2}	0.901	101.41	9.86×10^{-2}	0.999

Table S3. Intraparticle diffusion parameters for heavy metals and cationic dyes adsorption onto FSAH.

		k_{id1}/(mg g^{-1} min$^{-1/2}$)	k_{id2}/(mg g^{-1} min$^{-1/2}$)	C_1	C_2	R^2_1	R^2_2
FSAH	Pb^{2+}	4.4024	1.35×10^{-4}	73.1985	98.9923	0.921	0.999
	Cd^{2+}	3.6203	4.53×10^{-2}	63.0841	87.4502	0.909	0.978
	Cu^{2+}	6.2987	2.57×10^{-2}	31.0506	69.5148	0.889	0.971
	MB	7.8578	4.58×10^{-1}	45.2237	93.9044	0.905	0.972
	MG	11.4564	9.43×10^{-2}	29.2531	98.2433	0.932	0.991
	CV	10.8485	2.15×10^{-1}	29.9219	95.6726	0.917	0.962

Thermodynamic adsorption

Thermodynamic experiments can be evaluated the adsorption thermodynamic process of heavy metal ions and cationic dyes by FSAH based on Van't Hoff equation. The thermodynamic parameters of ΔG^{θ}, ΔH^{θ}, and ΔS^{θ} were calculated by following Equations (S7)–(S10) at different temperature (298.15, 308.15, and 318.15 K) [7,8]:

$$lnK^{\theta} = \frac{\Delta S^{\theta}}{R} - \frac{\Delta H^{\theta}}{RT} \quad \text{(S7)}$$

$$\Delta G^{\theta} = \Delta H^{\theta} - T\Delta S^{\theta} \quad \text{(S8)}$$

$$\Delta G^{\theta} =- RTlnK^{\theta} \quad \text{(S9)}$$

$$K^{\theta} = 1000 \times K_L\, M_A c^{\theta} \quad \text{(S10)}$$

where K^{θ}, R, K, T and M_A denotes the equilibrium constant, the gas constant (8.314 J·K^{-1}·mol^{-1}), the reaction rate constant, the absolute temperature (K) and the relative molecular mass of the adsorbate, g/mol, respectively.

Table S4. Thermodynamic parameters for the adsorption of heavy metal ions and dyes onto FSAH.

		T (K)	ΔS^{θ} (J (mol·K)$^{-1}$)	ΔH^{θ} (kJ mol^{-1})	ΔG^{θ} (kJ mol^{-1})	R^2
FSAH		318.15			-29.444	
		298.15			-21.119	
	Pb^{2+}	308.15	107.868	21.228	-22.307	0.938
		318.15			-23.385	
		298.15			-18.442	
	Cd^{2+}	308.15	195.571	39.867	-20.397	0.951
		318.15			-22.353	
		298.15			-17.469	
	Cu^{2+}	308.15	134.693	22.689	-18.816	0.981
		318.15			-20.163	
		298.15			-24.068	
	MB	308.15	272.271	57.109	-26.791	0.988
		318.15			-29.513	
		298.15			-21.515	
	MG	308.15	175.259	30.738	-23.268	0.933
		318.15			-25.021	
		298.15			-21.797	
	CV	308.15	121.355	14.385	-23.010	0.993
		318.15			-24.224	

Effect of salt cations

NaCl, KCl, and $CaCl_2$ was evaluate the adsorption effects of heavy metal ions (100 mg/L) and dyes (100 mg/L) in different concentrations (0 to 0.2 mol/L) at 298.15 K in Fig. S2. The removal efficiency of FSAH for heavy metal ions and cationic dyes significantly reduced with the salt ions concentration increased from 0 to 0.2 mol/L, which revealed a strong antagonistic effect on the adsorption of cationic dyes in the high salinity systems (0.2 mol/L). Ca^{2+} > Na^{+} > K^{+} and MG > MB > CV were the order of salt ions and cationic dyes adsorption interference, respectively. The results revealed that increasing salt ion concentration has a negative effect on electrostatic adsorption and inhibits cationic dyes adsorption [9]; The inhibition of physical adsorption on the surface of FSAH and the ability of functional groups to capture heavy metal ions, the divergence of the effects of these two coexisting ions was associated with the hydrated radii and electronegativity [10]. Furthermore, Ca^{2+} inhibits adsorption more effectively than Na^{+} because it facilitates adsorbate transport at saturated active pore sites and complexes with surface groups on the adsorbent [11–13]. Electrostatic adsorption was negatively affected by the ion impact caused by excessive salt content.

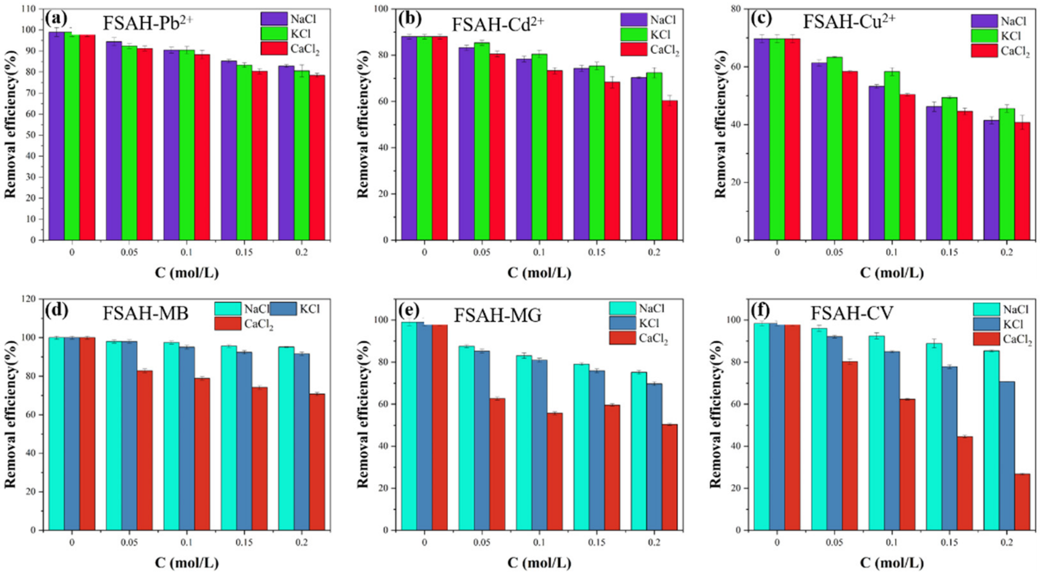

Figure S2. Effect of different salt ions on the adsorption capacity of FSAH for to adsorb Pb^{2+} (**a**), Cd^{2+} (**b**), Cu^{2+} (**c**), MB (**d**), MG (**e**), and CV (**f**).

Characterization

The surface morphology and distribution of element before and after adsorption of heavy metal ions was observed by scanning electron microscope (SEM, FEI Versa3D, Waltham, MA, USA) and energy dispersive spectroscopy (EDS-mapping) accessory. The range of 4000–400 cm^{-1} spectral peaks was recorded at 298.15 K before and after adsorption by Fourier-transform infrared spectroscopy (FT-IR, ThermoFisher Nicolet iS50, Waltham, MA, USA). BET method was determined to evaluate porosity and surface area (Micromeritics TriStar 3030, Waltham, MA, USA). The thermogravimetric test (TG, NETZSCH STA409PC, Überlingen, Germany) determined thermal stability. X-ray photoelectron spectroscopy (XPS, ThermoFisherr K-Alpha, Pittsburgh, PA, USA) was determined to analyze the composition of surface elemental. The atomic absorption spectrophotometer (AAS, Analytik Jena ZEEnit 700Q, Ilmenau OT Langewiesen, Germany) determined residual concentrations of Pb^{2+}, Cd^{2+}, and Cu^{2+} in solution. Residual cationic dyes concentration was detected by UV/Vis spectrophotometer (UVe5100B, Suzhou, China)

Batch equilibrium adsorption study

In batch adsorption research, Batch adsorption experiments were carried out in bottles containing heavy metal ions dye solutions (10 mL 100-2000 mg/L). 10 mg FSAH adsorbent was added to the above solutions and shaken in a water-bath thermostatic oscillator for 1 h. The concentration of the dye solution was determined at adsorption equilibrium. Equilibrium adsorption q_e is derived from Equation (S11).

$$q_e = \frac{(C_0 - C_e)V}{m} \tag{S11}$$

where C_0 (mg/L) and C_e (mg/L) are the initial and the residual concentration of dyes at time *t*, respectively, *V* (L) and *m* (g) are the volume of heavy metal ions and dye solution and the weight of the dried FSAH, respectively.

Table S5. EDS-mapping parameters of SA and FSAH, respectively.

SA				FSAH			
**Element**	**Weight %**	**Atomic %**	**Error %**	**Element**	**Weight %**	**Atomic %**	**Error %**
**C K**	32.49	38.71	6.30	**C K**	48.35	54.82	4.17
**N K**	0	0	0	**N K**	24.03	23.18	10.39
**O K**	48.34	47.52	6.93	**O K**	25.88	21.78	9.84
**Na K**	19.18	13.77	5.17	**Na K**	1.23	0.72	6.52

Table S6. Comparison with other adsorption materials.

**Adsorption Materials**	**Contaminant**	**q_m(mg/g)**	
Arg-MMT	Pb^{2+}	303.2	[14]
G-C_3N_4	Pb^{2+}	94.8	[15]
Fe_3O_4@TATA@ATA	Pb^{2+}	205.2	[16]
	MG	128.1	
PCRAC	Cu^{2+}	176.92	[17]
	Ni^{2+}	167.92	
NTA-β-CD-CS	MB	162.6	[18]
ENCC	Cu^{2+}	185	[19]
ECAA	Pb^{2+}	219.3	[20]
	Cu^{2+}	87.8	
Aam-MMT	Cu^{2+}	240	[21]
	Pb^{2+}	120	
PILS	Cd^{2+}	138.26	[22]
MPs	Pb^{2+}	689.3	[23]
	MG	682.3	
GFP	CV	254.2	[24]
POSS-Ni_xO_y-TiO_2	CV	848.5	[25]
Activated carbon-modified	CV	331.4	[26]
AAP polyHIPE	MB	791.17	[27]

References

1. Milani, S.A.; Karimi, M. Isotherm, kinetic and thermodynamic studies for Th(IV) sorption by amino group-functionalized titanosilicate from aqueous solutions. *Korean J. Chem. Eng.* **2017**, *34*, 1159-1169, doi:10.1007/s11814-016-0357-2.
2. Langmuir, I. The Constitution and Fundamental Properties of Solids and Liquids. Part I. Solids. *J. Am. Chem. Soc.* **1916**, *38*, 2221-2295, doi:10.1021/ja02268a002.
3. Freundlich, H. Über die Adsorption in Lösungen. *Z. Phys. Chem.* **1906**, *57U*, 385–470.
4. Chen, X.; Li, P.; Zeng, X.; Kang, Y.; Wang, J.; Xie, H.; Zhang, Y. Efficient adsorption of methylene blue by xanthan gum derivative modified hydroxyapatite. *Int. J. Biol. Macromol.* **2020**, *151*, 1040-1048, doi:10.1016/j.ijbiomac.2019.10.145.

5. Lagergren, S. Zur theorie der sogenannten adsorption geloster stoffe. *Kungliga Svenska Vetenskapsakademiens. Handlingar* **1898**, *24*, 1-39.
6. Ho, Y.S.; McKay, G. Sorption of dye from aqueous solution by peat. *Chem. Eng. J.* **1998**, *70*, 115-124.
7. Cestari, A.R.; Vieira, E.F.; Tavares, A.M.; Bruns, R.E. The removal of the indigo carmine dye from aqueous solutions using cross-linked chitosan: evaluation of adsorption thermodynamics using a full factorial design. *J. Hazard. Mater.* **2008**, *153*, 566-574, doi:10.1016/j.jhazmat.2007.08.092.
8. Zhang, N.; Zhang, H.; Li, R.; Xing, Y. Preparation and adsorption properties of citrate-crosslinked chitosan salt microspheres by microwave assisted method. *Int. J. Biol. Macromol.* **2020**, *152*, 1146-1156, doi:10.1016/j.ijbiomac.2019.10.203.
9. Dong, J.; Du, Y.; Duyu, R.; Shang, Y.; Zhang, S.; Han, R. Adsorption of copper ion from solution by polyethylenimine modified wheat straw. *Bioresour. Technol. Rep.* **2019**, *6*, 96-102, doi:10.1016/j.biteb.2019.02.011.
10. Wang; Xu, L.; Cheng, C.; Meng, Y.; Li, A. Preparation of new chelating fiber with waste PET as adsorbent for fast removal of Cu2+ and Ni2+ from water: Kinetic and equilibrium adsorption studies. *Chem. Eng. J.* **2012**, *193-194*, 31-38, doi:10.1016/j.cej.2012.03.070.
11. Yadav, S.; Asthana, A.; Singh, A.K.; Chakraborty, R.; Vidya, S.S.; Susan, M.; Carabineiro, S.A.C. Adsorption of cationic dyes, drugs and metal from aqueous solutions using a polymer composite of magnetic/beta-cyclodextrin/activated charcoal/Na alginate: Isotherm, kinetics and regeneration studies. *J. Hazard. Mater.* **2021**, *409*, 124840, doi:10.1016/j.jhazmat.2020.124840.
12. Chen, H.; Gao, B.; Li, H. Removal of sulfamethoxazole and ciprofloxacin from aqueous solutions by graphene oxide. *J. Hazard. Mater.* **2015**, *282*, 201-207, doi:10.1016/j.jhazmat.2014.03.063.
13. Chen, Y.; Lan, T.; Duan, L.; Wang, F.; Zhao, B.; Zhang, S.; Wei, W. Adsorptive Removal and Adsorption Kinetics of Fluoroquinolone by Nano-Hydroxyapatite. *PLoS ONE* **2015**, *10*, e0145025, doi:10.1371/journal.pone.0145025.
14. Chen, Y.; Wang, S.; Li, Y.; Liu, Y.; Chen, Y.; Wu, Y.; Zhang, J.; Li, H.; Peng, Z.; Xu, R.; et al. Adsorption of Pb(II) by tourmaline-montmorillonite composite in aqueous phase. *J. Colloid Interface Sci.* **2020**, *575*, 367-376, doi:10.1016/j.jcis.2020.04.110.
15. Hu, R.; Wang, X.; Dai, S.; Shao, D.; Hayat, T.; Alsaedi, A. Application of graphitic carbon nitride for the removal of Pb(II) and aniline from aqueous solutions. *Chem. Eng. J.* **2015**, *260*, 469-477, doi:10.1016/j.cej.2014.09.013.
16. Alqadami, A.A.; Naushad, M.; ZA, A.L.; Alsuhybani, M.; Algamdi, M. Excellent adsorptive performance of a new nanocomposite for removal of toxic Pb(II) from aqueous environment: Adsorption mechanism and modeling analysis. *J. Hazard. Mater.* **2020**, *389*, 121896, doi:10.1016/j.jhazmat.2019.121896.
17. Thanarasu, A.; Periyasamy, K.; Manickam Periyaraman, P.; Devaraj, T.; Velayutham, K.; Subramanian, S. Comparative studies on adsorption of dye and heavy metal ions from effluents using eco-friendly adsorbent. *Mater. Today Proc.* **2021**, *36*, 775-781, doi:10.1016/j.matpr.2020.07.001.
18. Usman, M.; Ahmed, A.; Yu, B.; Wang, S.; Shen, Y.; Cong, H. Simultaneous adsorption of heavy metals and organic dyes by beta-Cyclodextrin-Chitosan based cross-linked adsorbent. *Carbohydr. Polym.* **2021**, *255*, 117486, doi:10.1016/j.carbpol.2020.117486.
19. Sheikhi, A.; Safari, S.; Yang, H.; van de Ven, T.G.M. Copper Removal Using Electrosterically Stabilized Nanocrystalline Cellulose. *ACS Appl. Mater. Interfaces* **2015**, *7*, 11301-11308, doi:10.1021/acsami.5b01619.
20. Huang, Y.; Wang, Z. Preparation of composite aerogels based on sodium alginate, and its application in removal of Pb(2+)and Cu(2+) from water. *Int. J. Biol. Macromol.* **2018**, *107*, 741-747, doi:10.1016/j.ijbiomac.2017.09.057.
21. Ilgin, P.; Durak, H.; Gür, A. A Novel pH-Responsive p(AAm-co-METAC)/MMT Composite Hydrogel: Synthesis, Characterization and Its Absorption Performance on Heavy Metal İons. *Polym.-Plast. Technol. Eng.* **2015**, *54*, 603-615, doi:10.1080/03602559.2014.974189.
22. Ali, E.A.M.; Sayed, M.A.; Abdel-Rahman, T.M.A.; Hussein, R. Fungal remediation of Cd(ii) from wastewater using immobilization techniques. *RSC Adv.* **2021**, *11*, 4853-4863, doi:10.1039/d0ra08578b.
23. Lin, L.; Tang, S.; Wang, X.; Sun, X.; Yu, A. Hexabromocyclododecane alters malachite green and lead(II) adsorption behaviors onto polystyrene microplastics: Interaction mechanism and competitive effect. *Chemosphere* **2021**, *265*, 129079, doi:10.1016/j.chemosphere.2020.129079.
24. Saeed, A.; Sharif, M.; Iqbal, M. Application potential of grapefruit peel as dye sorbent: kinetics, equilibrium and mechanism of crystal violet adsorption. *J. Hazard. Mater.* **2010**, *179*, 564-572, doi:10.1016/j.jhazmat.2010.03.041.
25. Zhang, S.; Zhang, F.; Yang, M.; Fang, P. POSS modified NixOy-decorated TiO_2 nanosheets: Nanocomposites for adsorption and photocatalysis. *Appl. Surf. Sci.* **2021**, *566*, doi:10.1016/j.apsusc.2021.150604.
26. Ji, Q.; Li, H. High surface area activated carbon derived from chitin for efficient adsorption of Crystal Violet. *Diamond Relat. Mater.* **2021**, *118*, doi:10.1016/j.diamond.2021.108516.
27. Abebe, M.W.; Kim, H. Methylcellulose/tannic acid complex particles coated on alginate hydrogel scaffold via Pickering for removal of methylene blue from aqueous and quinoline from non-aqueous media. *Chemosphere* **2021**, *286*, 131597, doi:10.1016/j.chemosphere.2021.131597.
